# Influence of saliva on the sensory properties of US commercial smoke affected wines: Preliminary findings

**DOI:** 10.1002/fsn3.3954

**Published:** 2024-01-12

**Authors:** Victoria D. Paup, Maria L. Montero, Carolyn F. Ross, Jungmin Lee

**Affiliations:** ^1^ School of Food Science Washington State University Pullman Washington USA; ^2^ The National Food Lab Plymouth Minnesota USA; ^3^ National Center for Food Science and Technology (CITA) University of Costa Rica San José Costa Rica; ^4^ United States Department of Agriculture (USDA) – Agricultural Research Service (ARS) Horticultural Crops Production and Genetic Improvement Research Unit Corvallis Oregon USA

**Keywords:** e‐tongue, fire, saliva, smoke exposure, smoke taint

## Abstract

Previous research has suggested that the impact of smoke affected wines require human evaluation due to in‐mouth changes in perception, perhaps associated with saliva. Smoke affected wines (*n* = 36) from three major wine growing regions in the US were sourced from commercial wineries. A subset of these wines (*n* = 7) were evaluated by a consumer panel (*n* = 57) and electronic tongue (e‐tongue) to determine the influence of saliva in the sensory profile. Consumers assessed the wines for aroma and other sensory attributes, before and after individual saliva addition. Pooled saliva from consumers was used to treat all wines obtained (*n* = 36) and then analyzed using the e‐tongue. Results showed that saliva did not significantly alter the overall aroma, other than fruity or woody aroma liking by consumers (*p* > .05). However, the presence of saliva significantly lowered overall liking in both red and white wines that were affected by smoke (*p* ≤ .05). Consumers rated the subset of smoke affected wines below the “might purchase” category, indicating these wines were not considered acceptable by consumers. When individual pairs of smoke affected wines (before and after saliva additions) were assessed using the e‐tongue, the device was able to differentiate the pairs, validating potential usefulness to discern wine changes, though the discrimination indices were moderate to low (68.8% to 11.9%). Based on these data, in human ratings of the aroma and appearance of smoked affected wines, saliva decreased overall liking, and this was somewhat distinguishable by e‐tongue analysis.

## Graphical abstract

Consumers found a significant differences in overall liking and appearance liking after the saliva addition. The e‐tongue distinguished among most samples with added saliva and those same samples without saliva addition with low to moderate discrimination indices. This was based on the soluble profile of the samples from the taste map shifts in observation points and clear grouping by cluster analysis. This is the first paper to demonstrate e‐tongue’s potential for discriminating smoke affected wine with saliva addition.

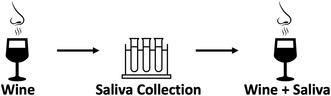

## 1 INTRODUCTION

Climate change has led to increased frequency, intensity, and duration of wildfires (Halofsky et al., 2020; Krstic et al., 2015). In 2020, California's grape industry had a crop loss payment of over $300 million (USDA Risk Management Agency, 2023) due to wildfire smoke exposure, resulting in significant unharvested grapes. In the states of Washington and Oregon, severe wildfire events caused grape loss payments of >$ 16 million and >$ 9.7 million, respectively (USDA Risk Management Agency, 2023).

Wildfire, agricultural burn, and smoke have been implicated in altering wine grape quality components, and ultimately the resulting wine (Krstic et al., 2015; Parker et al., 2013). Aroma, flavor, taste, and aftertaste sensory attributes such as smoky, burnt, dirty, ashy, medicinal, and ashtray have been associated with smoke affected wines (Coulter et al., 2022; De Vries et al., 2016; Mayr et al., 2014; Parker et al., 2012). These negative sensory profiles may result in unpalatable wines and significant financial losses to the winery (Kennison et al., 2007, 2008; Ristic et al., 2016).

Undesirable perceptions can be mixed due to cultivar responses, wine making practices, smoke composition, smoke intensity, smoke duration, smoke uptake, vineyard and winery mitigation efforts, and individual consumer panelist variables such as overall health, age, gender, taste and odor sensitivity, preferences, sip volume, oral microbiota, saliva composition, and production (Criado et al., 2022; De Vries et al., 2016; Genovese et al., 2015; Mayr et al., 2014; Parker et al., 2012, 2018, 2020; Perez‐Jimenez et al., 2023; Piombino et al., 2014; Ployon et al., 2017).

Previous sensory studies have reported wine aroma during consumption varies due to numerous factors, including types and quantity of released compounds during oral processing (Parker et al., 2012; Perez‐Jimenez et al., 2023; Ployon et al., 2017). Saliva is a complex fluid mostly consisting of water in combination with inorganic and organic electrolytes, proteins, and microorganisms (Parker et al., 2020; Ployon et al., 2017; Zhang et al., 2022). Due to chemical and physical changes that occur, saliva plays an important role in aroma release and perception of food and beverages (Munoz‐Gonzalez et al., 2014; Parker et al., 2012; Perez‐Jimenez et al., 2023; Ployon et al., 2017; Zhang et al., 2022). The enzymes and bacteria found in saliva and oral epithelial cells, in smoke affected wines, has been implicated in releasing smoke‐related compounds (Mayr et al., 2014; Parker et al., 2012, 2013, 2018). In addition to the variations in perception of individuals, tasting of smoke affected wines are further complicated by lingering flavors or aftertastes (Parker et al., 2012, 2013). A consumer study performed by Australian consumers (*n* = 82) regarding acceptance of smoke affected rosé (made from Pinot noir) found that those consumers did not significantly differentiate between a wine sample blended with 6.25% of smoke tainted wine and the non‐smoke affected control; however, differences were noted by consumers when the blend contained 12.5% and above smoke affected wine (The Australian Wine Research Institute, 2021). A small portion of those consumers did not perceive smoke affected rosé wines negatively.

Wine sensory panels of smoke affected wines have shown that human assessments are commingled with the multiple compounds that can be responsible for taint, with additive and synergistic effects making it challenging to decipher and link sensory results to individual chemical markers (Mayr et al., 2014; McKay et al., 2020; Parker et al., 2012, 2013). Instruments like the electronic tongue (e‐tongue) and electronic nose (e‐nose) have the potential to discern changes and differences between samples, as those assessments are not focused on specific compounds (Fuentes et al., 2020; Paup et al., 2021; Rodriguez‐Mendez et al., 2016; Ross, 2021; Summerson, Viejo, Pang, et al., 2021; Summerson, Viejo, Torrico, et al., 2021). Based on past research findings (Criado et al., 2022; Genovese et al., 2015; Mayr et al., 2014; Munoz‐Gonzalez et al., 2014; Parker et al., 2018, 2020; Ployon et al., 2017), postulations were made that saliva would release smoke taint related compounds from the suspect wine. In no previous studies have US wine consumers assessed the influence of saliva on acceptability of smoke affected commercial wines. Thus, the objective of this study was to examine the influence of human saliva on the perception of smoke affected wines by consumer sensory analysis and soluble organic and inorganic compounds by the e‐tongue.

## 2 MATERIALS AND METHODS

### 2.1 Wines evaluated by consumers and electronic tongue analyses

Commercial wines (*n* = 36; 2 to 3 bottles each) were provided by an industry cooperator. These wines had been identified by the industry cooperator internal sensory evaluations as having smoke taint and were sourced from several states across the USA, including California (CA, *n* = 29), Oregon (OR, *n* = 2), Washington (WA, *n* = 3), and state unknown (*n* = 2). All smoke affected wines were from the 2020 vintage. The identified samples were mostly red single‐varietal wines (34 of 36; two unknown red varietal examples with no varietal data provided) and two white single‐varietal wines. The varietal wines included Cabernet Sauvignon, Fiano, Malbec, Merlot, Mouvedre, Petite Verdot, Petite Syrah, Pinot gris, Pinot noir, and Syrah. A subset (*n* = 7, codes in Table 1) of this collection were used for consumer sensory evaluation, including both white wines (wines A and B). This subset was selected as having the most smoke taint, as determined by high selection rates for attributes associated with smoke affected wines, such as ashy, smoky, burnt, and rubber tire, by six expert panelists using Check‐All‐That‐Apply (CATA). Expert panelists were selected due to their extensive descriptive analysis experience and were highly educated in the sensory evaluation techniques. This expert panel was also used to determine the ideal time point for the saliva‐incubated wines to be evaluated. The expert panelists were provided with and instructed to rate the aroma intensity of saliva‐incubated wine every 30 s for 10 min, with the maximum intensity being observed after 3 min and 30 s. All 36 wines were used for the e‐tongue analysis portion. A commercial wine, Chateau St. Jean Soiree red wine 2014 (Kenwood, CA, USA; a blend) was used as a control for both the sensory panel and e‐tongue analysis. Control wine was evaluated by the same expert panel and had no smoke taint attributes detected.

**TABLE 1:** Consumer acceptance of smoke affected wines and control wine, coded with a different lowercase letter, and after treatment (NS = no saliva addition, S = saliva addition, and W = water addition) as either acceptable or unacceptable by the consumers (*n* = 57). Wines were assessed by appearance and aroma for acceptability. Comparisons were made between NS and S for % consumer acceptance and *p*‐values are presented.

Wine codes	Treatments	Consumer acceptance (%)	*p*‐Values
A	NS	42.1	.56
	S	28.1	
B	NS	36.8 a	.03
	S	21.0 b	
C	NS	91.2 a	<.0001
	S	35.1 b	
D	NS	82.5 a	<.0001
	S	26.3 b	
E	NS	86.0 a	<.0001
	S	33.3 b	
F	NS	89.5 a	<.0001
	S	45.6 b	
G	NS	78.9 a	<.0001
	S	31.6 b	
Control	NS	88.6 a	<.0001
	S	43.9 b	
	W	93.0 a	

*Note*: Wines A and B were white wines. Wines C through G were red wines. Control wine was a purchased red wine. Consumer unacceptance values not presented here, since they can be obtained by subtracting acceptance from 100%, and the statistical results were the same as what is presented here.

### 2.2 Initial panel evaluation

Participants, self‐reported as frequent wine consumers, answered a series of demographic and consumption habit questions. These questions included: gender, age, race, education, household income, smoking habits, household structure, wine consumption questions (as discussed in Bruwer et al., 2002, Bruwer & Li, 2007), self‐reported wine knowledge (Pomarici et al., 2017), a wine knowledge assessment (Schumaker et al., 2017), and smoked food consumption questions (Del Toro‐Gipson et al., 2021). A total of 57 participants conducted the evaluations: 70% were women, age ranged from 22 to 83 years (mean age = 41.5 years), 2% were current smokers, and 13% were past smokers. All panelists reported consuming smoked and/or grilled food; with 10% preferring high intensity, 67% medium intensity, and 23% low intensity smoke flavor. The use of human subjects for this study (# 19148‐002) was approved by Washington State University Institutional Review Board (Pullman, WA, USA). An informed consent was obtained from each subject prior to their participation in the study. At the evaluation completion, the consumer panelists received compensation for their time (i.e., gift card).

A decision was made that tasting will not be part of this project due to the COVID‐19 (coronavirus) pandemic related complexity and challenges to tasting saliva added wine (Ross, 2021). A subset of the commercial smoke affected wines (*n* = 7) was evaluated in a completely randomized design, before and after the addition of saliva (described below). To minimize fatigue, participants evaluated these wines over 3 days (3 wines on Day 1; 2 wines on Days 2 and 3). Participants were presented with either a red or white wine sample (20 mL) identified with a random 3‐digit code. The samples were poured 1 h prior to evaluation to allow equilibration. All samples were presented in standard ISO colorless wine glasses and covered with a glass petri dish. In this initial presentation, participants were first asked if the wine was acceptable or not (binary response) based on the aroma. The participants then assessed various appearance and aroma attributes of the wines. Specifically, participants rated their overall liking of the wine, the liking of appearance, liking of overall aroma, CATA for aromatic notes, and liking of aroma other than fruity or woody. The CATA question included terms that are commonly attributed to commercial wines (i.e., fruity, spicy, ethanol, burnt rubber/sulfur, leather, earthy, and woody) and then terms that were more specific to smoke exposed wines (i.e., smoky, burnt, ashy, medicinal, and smoked meats). A 9‐point hedonic scale was used to gather these data with 1 = dislike extremely to 9 = like extremely. For each coded sample, purchase intent was also asked along a 5‐point scale (1 = definitely would not buy to 5 = definitely would buy). Just about right (JAR; 1 = much too low, 3 = just about right and 5 = much too high) questions were asked for overall aroma, and aromas other than fruity or woody intensities. Smoky aroma liking was not specifically asked to reduce the potential of consumer bias, rather it was determined by the attributes that remained when two of the most common attributes, fruity or woody, were not considered. Comments were also collected.

### 2.3 Saliva collection and panel evaluation after saliva addition

To collect the saliva, participants were instructed to chew on a small piece of parafilm (3.81 × 2.54 cm) for 10 min and expectorate into a 50 mL vial. During testing the collected saliva was stored in a 37°C water bath for maximum 30 min to limit compositional changes (Mayr et al., 2014). The consumers' own saliva (3 mL) was then added back into a 20 mL wine sample with slight modifications as described in Buettner (2002) and Genovese et al. (2009), and then mixed carefully to minimize foaming. Leftover saliva was pooled for the e‐tongue portion of this work (described below). After a 3.5 min incubation at 22°C, as determined by the expert panel, the wine‐saliva mixture was presented back to the participant. Participants were first asked if the wine was acceptable or not based on the aroma. The participant continued to evaluate the aroma and answered the same questions as described above for the wine with no saliva added. These questions included overall liking of the wine, the liking of appearance, liking of overall aroma, CATA for aromatic attributes, and liking of aroma other than fruity or woody. For each coded sample, purchase intent was also asked. Just about right (JAR; 1 = much too low, 3 = just about right and 5 = much too high) questions were asked for overall aroma, and aroma other than fruity or woody intensities. Comments were also collected.

Unused saliva from the panelists (*n* = 57) was pooled to produce ~1 L of saliva which was then stored at −80°C until it was utilized for treating samples in the e‐tongue portion of the study. Following sensory testing, the leftover wines were immediately flushed with nitrogen, recapped, and stored at 4°C until sample preparation for e‐tongue analysis. This was done due to the limited quantity supplied by the industry cooperator.

### 2.4 E‐tongue analysis

Samples were analyzed using a potentiometric e‐tongue (Astree® II electronic tongue unit, Alpha MOS®, Toulouse, France) equipped with 48‐well auto sampler and Set #6 sensors. The e‐tongue utilized seven cross‐selective taste sensors, a reference electrode (Ag/AgCl), and the AlphaSoft software (version 12). Instrument preparation, conditioning, calibration, and diagnostics were followed according to manufacturers' procedures, as previously described (Diako et al., 2017; Paup et al., 2019). The position of each treatment sample in the sequence was randomized to prevent measurement bias.

All 36 smoke affected wines and controls were tested by the e‐tongue, before and after composite saliva addition. An additional control, with a volume of distilled water equal to the saliva addition of treatment samples (3 mL; 37°C), was included and handled identically as saliva treated wines. The number of smoke affected wines available prevented water spiking (dilution) from being conducted on each sample. For the saliva (spiked) samples, 3 mL of thawed saliva was added to each 20 mL smoked wine sample and allowed to incubate for 3 min immediately prior to e‐tongue analysis. The control functioned as a reference to allow comparison between runs. All samples were analyzed in triplicate.

### 2.5 Data analysis

Statistical software, XLSTAT 2022 2.1 (Addinsoft, New York, USA), was used for all sensory data analyses. A two‐way analysis of variance (ANOVA) was conducted to evaluate the consumer liking results of different sensory attributes: overall liking, appearance, initial aroma, aromas other than fruity or woody, and purchase intent. The main factors in the ANOVA were wine (W) and saliva treatment (S). The W and S interaction (W × S) was also analyzed. Liking scores and the purchase intent means were separated with Tukey's Honestly Significant Difference (HSD) test. Consumer acceptability of the wine subset was determined by the frequency of mentioning if the wine was considered acceptable. The results were presented as percentages. The JAR scale results for the attributes initial aroma and aromas other than fruity or woody were interpreted with penalty analysis for each of the wines subset and the controls with and without the addition of saliva. Only wines A, C, and F results are presented as representative samples from the consumer portion of this study due to the similar trends observed to the other samples tested. These three wines were considered highly smoky by the expert panels.

E‐tongue data were analyzed using AlphaSoft version 20 (Alpha MOS, Toulouse, France). The average of triplicate sensor data not exceeding 15% RSD (relative standard deviation) were used in principal components analysis (PCA). Agglomerative Hierarchical Cluster analysis was completed using XLSTAT 2020 utilizing Euclidean distances and Ward's method for agglomeration. The discrimination index (DI) indicated how well the e‐tongue discriminated among the samples, taking into consideration the distance and overlap among sample triangles. Strongest (maximum) discrimination between samples is indicated by a discrimination index of 100% and no discrimination below 0% per manufacturer recommendation.

## 3 RESULTS AND DISCUSSION

### 3.1 Consumers' acceptability of the sensory profile of smoke affected wines

**FIGURE 1:**
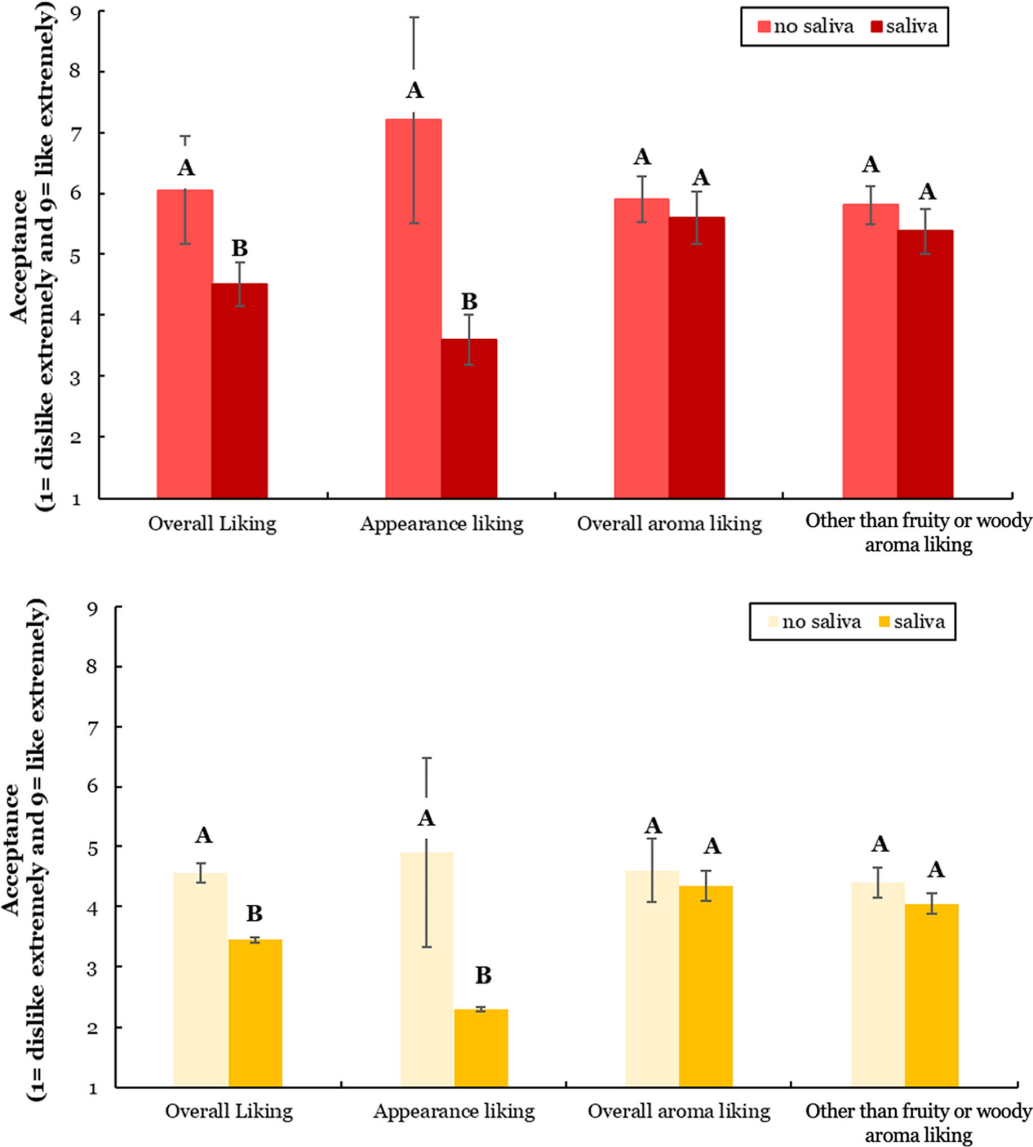
Comparison of the subset of red wines (top bar graph; 5 reds) and white wines (bottom bar graph; 2 whites), without and with added saliva for overall acceptance, appearance liking, overall aroma liking, and other than fruity or woody aroma liking rated by consumers (*n* = 57). All values were assessed along a 9‐point hedonic scale (1 = dislike extremely, 5 = neither dislike nor like, to 9 = like extremely; *y*‐axis). A different uppercase letter within a pair of bars indicates a significant difference (*p* ≤ .05). Error bars are standard deviations.

Overall liking and appearance liking by consumers significantly (*p* ≤ .05) decreased with the addition of saliva (Figure 1). After saliva was added, consumer liking was not significantly different between both smoke affected red and white wines in overall aroma liking or liking of aromas other than fruity (Figure 1). Consumer acceptance (based on aroma and appearance combined) of all smoke affected and control wine decreased by 15.8% (wine B) to 56.2% (wine D) with the addition of saliva (Table 1), except for wine A (same trend, but not significant). These findings showed saliva negatively influenced liking scores of the smoke affected wine, signifying efforts to reduce the perception of smoke need to consider in‐mouth release of smoke compounds (Parker et al., 2012, 2013). At the same time, water added to a control wine increased their acceptability, which suggested changes perceived by consumers were beyond volumetric dilution. This was also observed by Munoz‐Gonzalez et al. (2014) in aroma analysis contrasting saliva or water additions. Both white wines (wines A and B) had a greater number of consumers who found them unacceptable compared to the red wines, although acceptability significantly decreased with saliva addition for wine B. Unfortunately, red or white wine preference was not part of the panelist questionnaire, so it is unknown if consumers preferred red wines in general.

One factor influencing the appearance evaluation may have been the visual difference due to the saliva addition in a small number of samples, possibly resulting from the precipitation of wine phenolics and salivary proteins (Rinaldi et al., 2012). Precipitation has been reported to affect wine aroma assessment (Williams et al., 1984). The appearance change from the saliva addition was not anticipated since precipitation was not noted during the expert panel assessment, so actions to obscure samples' appearance were not performed for the consumers study (e.g., dark colored glasses, booth light adjustment, or decanting mixture before serving). This confirms that differences in human saliva can vary in their potential to impact wine tasting due to in‐mouth precipitation by saliva components or conditions (Brandao et al., 2020).

Individual wines and saliva treatment (as main effects) were significant factors influencing overall liking and other than fruity or woody liking (Table 2). There were also significant interactions between the individual wines and saliva treatment (W × S) influencing appearance liking and purchase intent suggesting that the wine was also a source of variation in the results.

**TABLE 2:** F‐ratios from analysis of variance by the consumer sensory panel (*n* = 57) assessment of commercial smoke affected wines, examining the influence of the individual wines (W), saliva treatment (S), and the interaction between individual wines and saliva treatment (W × S).

Source	df	Overall liking	Appearance liking	Overall aroma liking	Other than fruity or woody liking	Purchase intent
W	8	12.85**	38.63**	21.40**	21.93**	16.47**
S	1	132.24**	1038.53**	3.62	4.49*	97.46**
W × S	6	1.77	18.08**	0.95	0.77	5.58**

* *p* ≤ .05.

** *p ≤* .0001.

The liking scores were separated for individual wines and the control in Table 3. Before saliva addition, smoke affected white wines (wines A and B) were scored with significantly lower values for overall liking, appearance liking, overall aroma liking, aromas other than fruity or woody liking, and purchase intent compared to red wines. All smoke affected red wines (as‐is, wines C through F) were not significantly different in their scores for overall liking, appearance liking, overall aroma liking, other than fruity or woody liking, and purchase intent. Most red wines (as‐is except wine G; including control) had a purchase intent score that exceeded 3.0, where 3.0 rating equaled “may or may not purchase.” Purchase intent for all wines (as‐is) did not reach “might purchase” category, indicating the possible problems these wines could have with retail sales.

**TABLE 3:** Means of the liking scores along a 9‐point scale (1 = dislike extremely to 9 = like extremely) and purchase intent of a consumer sensory panel (*n* = 57). Tukey HSD results for the smoke affected commercial wines and control wine treated with saliva (NS = no saliva addition, control 1, and S = saliva addition, control 2) or water (W = water addition, control 3) are indicated with a different lowercase letter (*p* ≤ .05) within a column. Standard deviation provided after mean.

Wine codes	Overall liking		Appearance liking		Overall aroma liking		Other than fruity or woody liking		Purchase intent	
	NS	S or W	NS	S or W	NS	S or W	NS	S or W	NS	S or W
A	4.4 ± 2.34 a	3.7 ± 2.21 ab	6.5 ± 1.82 a	2.6 ± 1.85 a	3.8 ± 2.22 a	4.1 ± 2.17 a	3.8 ± 2.01 a	3.9 ± 2.00 a	2.0 ± 1.04 a	1.9 ± 1.10 a
B	4.7 ± 2.21 a	3.6 ± 2.32 a	3.3 ± 2.00 b	2.6 ± 1.73 a	4.7 ± 2.26 b	4.7 ± 2.19 ab	4.3 ± 2.14 a	4.2 ± 2.13 ab	2.1 ± 1.15 a	2.0 ± 1.09 a
C	6.7 ± 1.67 b	4.7 ± 2.39 bcd	7.4 ± 1.36 cd	3.4 ± 2.33 ab	6.4 ± 1.76 c	6.1 ± 1.90 de	6.1 ± 1.85 b	5.7 ± 1.64 cde	3.4 ± 1.08 b	2.5 ± 1.21 b
D	5.9 ± 2.23 b	4.0 ± 2.18 abc	6.8 ± 1.76 ad	2.7 ± 1.73 a	5.7 ± 2.25 c	5.7 ± 1.76 cde	5.4 ± 2.12 b	5.4 ± 1.76 cde	3.1 ± 1.27 b	2.2 ± 1.10 ab
E	6.0 ± 1.91b	4.3 ± 2.23 abcd	7.8 ± 0.82 c	3.6 ± 2.21 b	6.0 ± 1.89 c	5.5 ± 2.08 bcd	5.7 ± 1.91 b	5.1 ± 1.98 bcd	3.2 ± 1.13b	2.1 ± 1.12 ab
F	6.5 ± 1.79 b	5.1 ± 2.21 d	7.8 ± 1.14 c	4.1 ± 2.45 b	6.6 ± 1.78 c	6.3 ± 1.76 de	6.1 ± 1.78 b	5.9 ± 1.78 de	3.5 ± 1.17 b	2.6 ± 1.27 b
G	5.8 ± 2.02 b	4.3 ± 2.12 abcd	7.5 ± 1.09 cd	3.3 ± 2.01 ab	5.6 ± 2.10 c	4.9 ± 2.14 abc	5.3 ± 2.00 b	4.8 ± 1.91 abc	3.0 ± 1.18 b	2.1 ± 1.06 ab
Control 1	6.4 ± 1.49 b	‐	7.6 ± 1.07 c	‐	6.3 ± 1.54 c	‐	5.8 ± 1.60 b	‐	3.4 ± 0.91 b	‐
Control 2	‐	4.8 ± 1.53 cd	‐	3.7 ± 1.26 b	‐	5.8 ± 1.63 cde	‐	5.4 ± 1.80 cde	‐	2.4 ± 1.08 ab
Control 3	‐	6.8 ± 2.31 e	‐	7.3 ± 2.23 c	‐	6.6 ± 1.99 e	‐	6.1 ± 1.77 e	‐	3.6 ± 1.22 c

*Note*: Wines A and B were white wines. Wines C through G were red wines. Controls 1–3 utilized a purchased red wine.

Overall liking scores for all the wines (smoke affected and control) significantly (*p* ≤ .05) decreased with the addition of saliva (Table 3). Water addition to the control wines (Control 1 vs. Control 3, in Table 3) increased overall liking, appearance liking, overall aroma liking, other than fruity or woody aroma liking, and purchase intent. In general, the interaction between saliva addition and wine type decreased appearance liking scores and purchase intent ratings for all smoke affected wines. Other than fruity or woody aroma liking results were higher for as‐is red wines than white wines. Purchase intent scores might have been driven by wine appearance and wine type (red vs. white) as the addition of saliva decreased these values in red wines; these relationships should be investigated further.

Few studies have been conducted on consumer acceptance and purchase intent of smoke affected wines. In an Australian study, most consumers (*n* = 82) disliked smoke affected rosé (The Australian Wine Research Institute, 2021). Work on (before and after) smoke exposure of grapes from a pine and selected vegetation fire (commonly found plants in Western Cape of South Africa) has shown smoke affected wine ended up with “burnt rubber” taint in Cabernet Sauvignon wine compared to control (not smoke affected) (De Vries et al., 2016). Fewer studies have explored the influence of saliva on consumer acceptance of smoke‐affected wines.

To discuss JAR and the penalty analysis (mean drop) results, three smoke affected wines (wines A‐ Pinot gris, C‐ Pinot noir, and F‐ Merlot, range of style) from the consumer testing were selected as representative samples (Table 4). From the JAR results, most of the consumers thought wine A (Pinot gris) had too high an initial aroma and aromas other than fruity or woody. Most consumers thought wine A was far from JAR, with only 5.26% finding wine A to have too little initial aroma and 63.13% finding it to have too much aroma. Through penalty analysis, it was found that for wine A, having too little initial aroma was less penalized, causing only a 1.17 (along a 9‐point scale) drop in liking. This was contrasted with having too much initial aroma, which caused a larger drop in liking mean value (2.69). Therefore, having a decrease in overall aroma, through mitigation efforts, may improve quality. Additionally, the inclusion of saliva did not significantly affect initial overall aroma liking, nor liking of aromas other than fruity or woody liking scores. Despite the lack of significant changes in liking scores, after the addition of saliva, the number of consumers that thought that wine A had too little aroma decreased. These results indicate saliva may not have a significant impact on aroma liking in white wine.

**TABLE 4:** Percentage of consumer responses and mean drop values from penalty analysis of the overall liking scores when the initial aroma, and other than fruity or woody aroma intensity were included in the analysis. Wines A (Pinot gris), C (Pinot noir), and F (Merlot) were selected as representative samples from the consumer testing.

Wine codes	Variables	Levels	Treatments	% consumer responses that were not JAR	Mean drop in overall liking scores	*p*‐Value
A	Initial aroma	Not enough	NS	5.26	1.17	<.0001
			S	8.77	0.90	.14
		Too much	NS	63.16	2.69	<.0001
			S	56.14	0.92	.14
	Aromas other than fruity or woody	Not enough	NS	14.04	0.41	.03
			S	12.28	0.19	.20
		Too much	NS	59.65	1.80	.03
			S	50.88	0.92	.20
C	Initial aroma	Not enough	NS	12.28	0.98	.001
			S	12.28	0.46	.02
		Too much	NS	28.07	1.64	.001
			S	19.30	2.36	.02
	Aromas other than fruity or woody	Not enough	NS	17.54	0.80	.01
			S	19.30	1.08	.02
		Too much	NS	29.82	1.26	.01
			S	21.05	1.85	.02
F	Initial aroma	Not enough	NS	5.26	3.89	<.0001
			S	8.77	1.09	.02
		Too much	NS	31.58	1.61	<.0001
			S	19.30	1.76	.02
	Aromas other than fruity or woody	Not enough	NS	8.77	0.75	.001
			S	12.28	0.82	.01
		Too much	NS	33.33	1.73	.001
			S	19.30	1.99	.01

Most consumers thought wines C (Pinot noir) and F (Merlot) had JAR aroma intensity, and the consumers who felt that the aroma of the red wine was JAR increased following the saliva addition. For wine C, penalty analysis determined that not having enough initial aroma caused a drop in aroma liking of 0.98 (without saliva) and 0.46 (with saliva added). However, having too much aroma caused a larger decrease in liking, which increased further after saliva addition (change in mean drop from 1.64 to 2.36). This indicated that saliva addition had a larger impact on liking associated with having too much aroma. In general, for the smoke affected wines tested, having too much aroma had a greater impact on the aroma liking of the samples than having too little aroma impact. These results indicate that reducing the overall aroma of a smoke affected wine may improve acceptability, even if other sensory notes are reduced as well.

Previous studies have reported mixed results on release and removal of smoke compounds during winemaking. Some of these processes, including processing enzymes to release, and removal by charcoal and fining, successfully decrease the smoke taint that ends up in the bottle (McKay et al., 2020; Summerson, Viejo, Torrico, et al., 2021). However, the consequences of such actions to wine quality (i.e., removing desirable compounds) need to be studied further.

### 3.2 E‐tongue analysis of smoke affected wines

**FIGURE 2:**
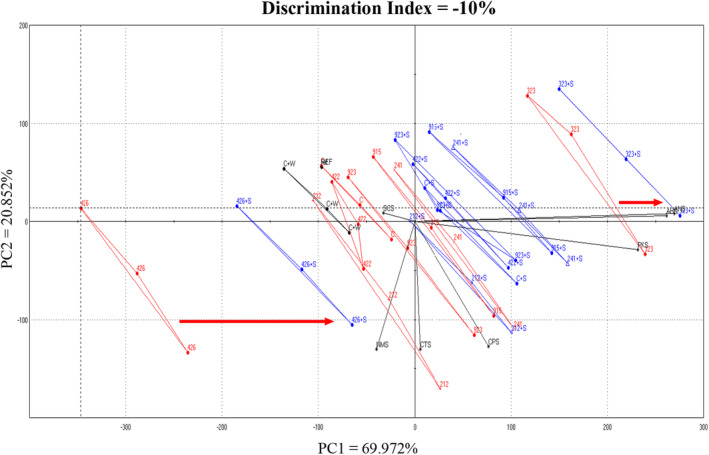
Taste map from the electronic tongue data for the subset of smoke affected commercial wines evaluated by consumers. Wines without saliva are shown in red, and wines with added composite saliva are in blue and labeled with +S. The discrimination index was −10%, but these values increased when individual pairs were compared (Table 5). For simplicity, only two pairs (as‐is vs. saliva added wine) were labeled with a red arrow (**→**) to show the shift to the right, but all wines (subset of seven and control) shifted to the right once saliva was added. Three‐digit codes in the figures were randomly assigned for e‐tongue data collection, except for C (control). Water addition indicated by +W.

**FIGURE 3:**
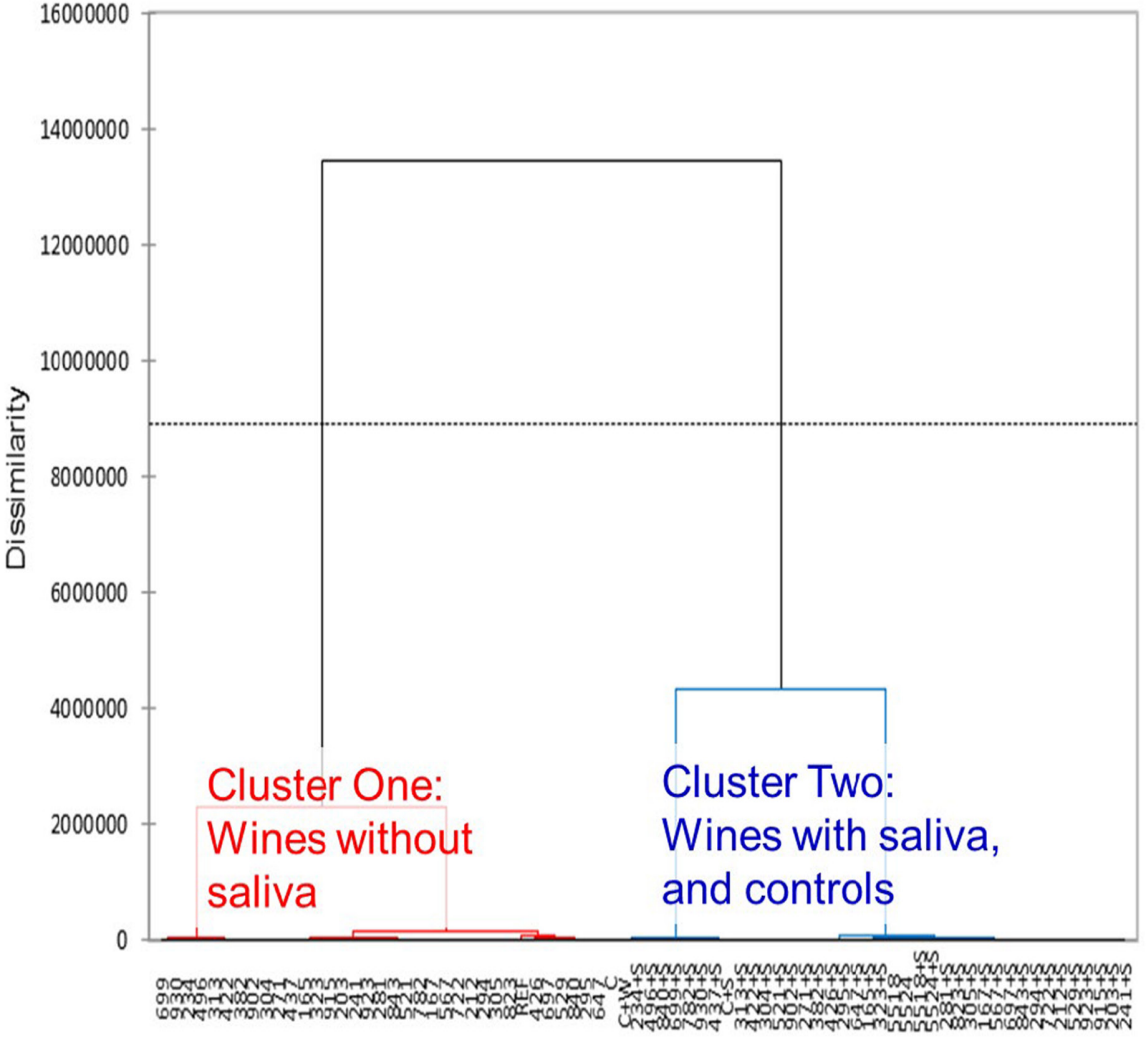
Agglomerative hierarchical cluster analysis of the electronic tongue (e‐tongue) results based on dissimilarity (*p* ≤ .05), clearly grouped results into two clusters. Clustering was mainly based on the whether or not saliva was added, with the exception of the controls which were more similar to the saliva added samples. Composite saliva added samples were labeled with +S. Codes on the *x*‐axis were random codes used for e‐tongue data collection.

For the 36 wines, when paired individual wine samples between no saliva added (NS) and saliva added (S) were compared by the e‐tongue, the discrimination values ranged from 11.9% (wine B) to 68.8% (wine A) and all wines were significantly different from each other (average was 38.0%; data not shown). The two extreme discrimination values were both white wines, which were among the seven wines in the consumer study; wine B (Fiano) had the lowest and wine A (Pinot gris) had the highest discrimination index. The e‐tongue results for the seven smoke affected wines and control from the consumer panel testing is summarized in Table 5. Only wines A (Pinot gris) and C (Pinot noir) had moderate discrimination index values, despite the shift seen in Figure 2 and clustering seen in Figure 3. When control wine with saliva addition and control wine with water addition were compared, the discrimination index was the highest at 71.3%, further supporting the changes was not due to simple dilution (Table 5).

**TABLE 5:** Pattern discrimination indices and distance based on *Astree* electronic tongue results for smoke affected wines that were selected for the consumer sensory test and controls, with and without saliva or water addition. All paired comparisons were significantly different, where *p*‐values were below .001.

Wine codes	Comparison made	Distances	Pattern discrimination index (%)
A	NS vs S	218.1	68.8
B	NS vs. S	59.6	11.9
C	NS vs. S	125.5	58.4
D	NS vs. S	89.3	25.1
E	NS vs. S	107.5	29.8
F	NS vs. S	80.5	20.2
G	NS vs. S	92.8	25.6
Control	NS vs. S	120.1	56.7
Control	NS vs. W	47.1	23.6
Control	S vs. W	160.7	71.3

*Note*: Wines A and B were white wines. Wines C through G were red wines. Control was a purchased red wine.

Abbreviations: NS, saliva addition; S, saliva addition; W, water addition.

When e‐tongue results from all 37 wine/wine and saliva combination samples (smoke affected wines and control wine) were pooled, the e‐tongue was unable to discriminate among these samples (discrimination index of −565%; data not shown). Results overlapped and no clear separation was observed based on PC 1 and PC 2 (variation of 89% explained) due to the saliva addition. A subset of wine samples (*n* = 7 and control) used for the consumer panel evaluation were extracted to better illustrate the results (Figure 2). A clear shift from the left to the right in the PCA placement was observed due to saliva addition. By only including the results of the wine subset used for the consumer testing, the discrimination index increased substantially to −10%, but still well below a high discrimination index. A 91% variation between the samples were explained by the PC 1 and PC 2. Control wine after saliva addition followed the same trend of shifting from left to right, but the addition of water shifted the results in the opposite direction, which indicated that the shift due to saliva addition was not from dilution, confirming the consumer panel acceptance results previously presented.

Agglomerative hierarchical cluster analysis was performed using the e‐tongue results, and there were two clear significant clusters (Figure 3, clusters 1 and 2), which were based on the presence or absence of saliva. All controls (as‐is, saliva addition, and water addition) were grouped with smoke affected wines with saliva addition (cluster 2), which suggests that that saliva addition altered the smoke characteristics and made these wines more similar to control wines. Due to the lack of discrimination observed when all 37 wines with and without the addition of saliva based on PC1 and PC2, as discussed previously, the observed clustering was most likely based on PC 3. If the e‐tongue comparison was performed on smoke affected wines made from a single grape cultivar, it is possible the overall discrimination index would be higher between no saliva and saliva addition, by reducing the complexity of comparing numerous base varietal characteristics (Gutierrez et al., 2010; Rodriguez‐Mendez et al., 2016).

Since this is the first work on smoke affected wine and changes due to saliva measured by e‐tongue, it is challenging to discuss this work among other findings. There are two studies that used the e‐nose to examine smoke affected wine with no saliva addition (Summerson, Viejo, Pang, et al., 2021; Summerson, Viejo, Torrico, et al., 2021). However, it must be considered that the two instruments (e‐tongue vs. e‐nose) use different sensors since they are intended to mimic different human senses, taste versus smell, respectively. The e‐tongue has been successfully used to monitor known wine fault compound development (acetic acid, methanol, ethyl acetate, 4‐ethylphenol, 4‐ethylguiacol, etc.) during fermentation, oak barrel aging, and final bottled wines (Paup et al., 2021; Rodriguez‐Mendez et al., 2016), so it is not surprising e‐tongue was able to discriminate between a saliva addition.

### 3.3 Limitations and future studies

Our findings demonstrated that e‐tongue has potential despite its limitations, which include the initial cost of the instrument, specialized training needed, drift due to temperature, constant calibration required, and challenges to link consumer acceptance with e‐tongue response (Rodriguez‐Mendez et al., 2016; Ross, 2021). Again, no work to date has been performed utilizing e‐tongue after saliva addition to wines. However, there was one recent study on how e‐tongue was able to discriminate between healthy and unhealthy individuals' saliva based on the presence (or absence) of oral cavity cancer (Braz et al., 2022).

No additional information regarding these smoke affected wines was provided by the industry, so it is unknown if any mitigation efforts were attempted. Based on the panel aroma and e‐tongue results, it appears volatile and soluble compounds in smoke affected wines were both impacted by saliva. Additional work is needed on the saliva released smoke compounds like concentration released, perception thresholds, and the ability to discriminate these compounds (Parker et al., 2020). Further sensory work should explore how in‐mouth precipitation in smoke affected wine impacts its perception.

It would be interesting to study aftertaste measurements with the e‐tongue (Ross, 2021) since smoke affected wines have been reported to have an aftertaste and carry over effects (Parker et al., 2012). E‐tongue experiments with smoke affected indicator compounds would help establish the limits of detection and the scale of time‐release for these compounds. It would be also worthwhile to study smoke affected wines with e‐tongue using altered wine compositions like varying ethanol amounts, pH, residual sugar, and any other variables that might alter perception of smoke related compounds (Mayr et al., 2014; Rinaldi et al., 2012). Although some parallel work has been done on that topic with e‐nose (Summerson, Viejo, Pang, et al., 2021; Summerson, Viejo, Torrico, et al., 2021). Research on in‐vitro smoke compound release conditions that better mimic in‐mouth release would also help our understanding of these compounds and linking chemical analysis to sensory results.

## 4 CONCLUSIONS

Consumers did not find a significant difference in overall aroma liking or liking of aromas other than fruity or woody between as‐is smoke affected wine and saliva added wine, but there were significant differences in overall liking and appearance liking after the saliva addition. The e‐tongue distinguished among most samples with added saliva and those same samples without saliva addition with low to moderate discrimination indices. This was based on the soluble profile of the samples from the taste map shifts in observation points and clear grouping by cluster analysis. This is the first paper to demonstrate e‐tongue's potential for discriminating smoke affected wine with saliva addition. Further research is needed to fully understand the implications saliva has on the soluble and volatile profiles of a smoke affected wine, human saliva variability, in‐mouth precipitation, and then to link this type of analysis to smoke affected wine compounds.

## AUTHOR CONTRIBUTIONS

**Victoria D. Paup:** Conceptualization (supporting); data curation (lead); formal analysis (lead); investigation (equal); methodology (equal); writing – original draft (equal); writing – review and editing (equal). **Maria L. Montero:** Formal analysis (supporting); writing – original draft (supporting); writing – review and editing (supporting). **Carolyn F. Ross:** Conceptualization (equal); data curation (equal); formal analysis (equal); funding acquisition (lead); investigation (lead); methodology (lead); project administration (lead); resources (equal); software (equal); supervision (lead); visualization (equal); writing – original draft (equal); writing – review and editing (equal). **Jungmin Lee:** Conceptualization (equal); formal analysis (supporting); funding acquisition (lead); investigation (equal); methodology (supporting); project administration (supporting); resources (equal); visualization (equal); writing – original draft (lead); writing – review and editing (lead).

## CONFLICT OF INTEREST STATEMENT

The authors declare no conflicts of interest.

## Data Availability

Data will be made available upon request.
